# Aminomethylphosphonic acid inhibits growth and metastasis of human prostate cancer in an orthotopic xenograft mouse model

**DOI:** 10.18632/oncotarget.7055

**Published:** 2016-01-28

**Authors:** Keshab Raj Parajuli, Qiuyang Zhang, Sen Liu, Zongbing You

**Affiliations:** ^1^ Departments of Structural & Cellular Biology, Tulane University, New Orleans, Louisiana 70112, USA; ^2^ Department of Orthopaedic Surgery, Tulane University, New Orleans, Louisiana 70112, USA; ^3^ Tulane Cancer Center and Louisiana Cancer Research Consortium, Tulane University, New Orleans, Louisiana 70112, USA; ^4^ Tulane Center for Stem Cell Research and Regenerative Medicine, Tulane University, New Orleans, Louisiana 70112, USA; ^5^ Tulane Center for Aging, Tulane University, New Orleans, Louisiana 70112, USA

**Keywords:** aminomethylphosphonic acid, glyphosate, prostate cancer, metastasis, orthotopic xenograft mouse model

## Abstract

Aminomethylphosphonic acid (AMPA) has been shown to inhibit prostate cancer cell growth *in vitro*. The purpose of the present study was to determine if AMPA could inhibit growth and metastasis of prostate cancer *in vivo*. Human prostate cancer PC-3-LacZ-luciferase cells were implanted into the ventral lateral lobes of the prostate in 39 athymic Nu/Nu nude male mice. Seven days later, mice were randomized into the control group (*n* = 14, treated intraperitoneally with phosphate buffered saline), low dose group (*n* = 10, treated intraperitoneally with AMPA at 400 mg/kg body weight/day), and high dose group (*n* = 15, treated intraperitoneally with AMPA at 800 mg/kg body weight/day). Tumor growth and metastasis were examined every 4-7 days by bioluminescence imaging of live mice. We found that AMPA treatment significantly inhibited growth and metastasis of orthotopic xenograft prostate tumors and prolonged the survival time of the mice. AMPA treatment decreased expression of *BIRC2* and activated caspase 3, leading to increased apoptosis in the prostate tumors. AMPA treatment decreased expression of cyclin D1. AMPA treatment also reduced angiogenesis in the prostate tumors. Taken together, these results demonstrate that AMPA can inhibit prostate cancer growth and metastasis, suggesting that AMPA may be developed into a therapeutic agent for the treatment of prostate cancer.

## INTRODUCTION

Aminomethylphosphonic acid (AMPA) is the primary degradation product of glyphosate [N-(phosphonomethyl) glycine]. Glyphosate is a broad-spectrum herbicide used to kill weeds [1]. Glyphosate can be converted into AMPA by microorganisms in the environment or animal bodies [2]. AMPA has no significant toxicity in acute, subchronic and chronic animal studies, nor any genotoxicity, teratogenicity or carcinogenicity [3].

Cancer cells undergo specific metabolic reprogramming to sustain cell survival and proliferation [4, 5]. In addition to energy consumption, cancer cells also accumulate cellular components, including nucleic acids, proteins, and lipids, as well as important cofactors [6]. It has been shown that the rapidly-proliferating cancer cells consume excessive glycine compared to the rapidly-proliferating non-transformed cells. Silencing hydroxymethyltransferase (SHMT, an enzyme for conversion of serine into glycine) expression and deprivation of extracellular glycine can inhibit proliferation of the cancer cells [7]. AMPA and glyphosate are analogs of glycine, which can inhibit the enzyme activities of SHMT [8]. We have previously shown that glyphosate and AMPA can inhibit proliferation and promote apoptosis in human prostate cancer cells, but not in normal cells *in vitro* [9, 10]. In the present study, our objective was to determine if AMPA could inhibit prostate cancer growth and metastasis *in vivo*.

## RESULTS

### AMPA treatment inhibits growth of orthotopically xenografted human prostate cancer in athymic nude mice

**Figure 1:**
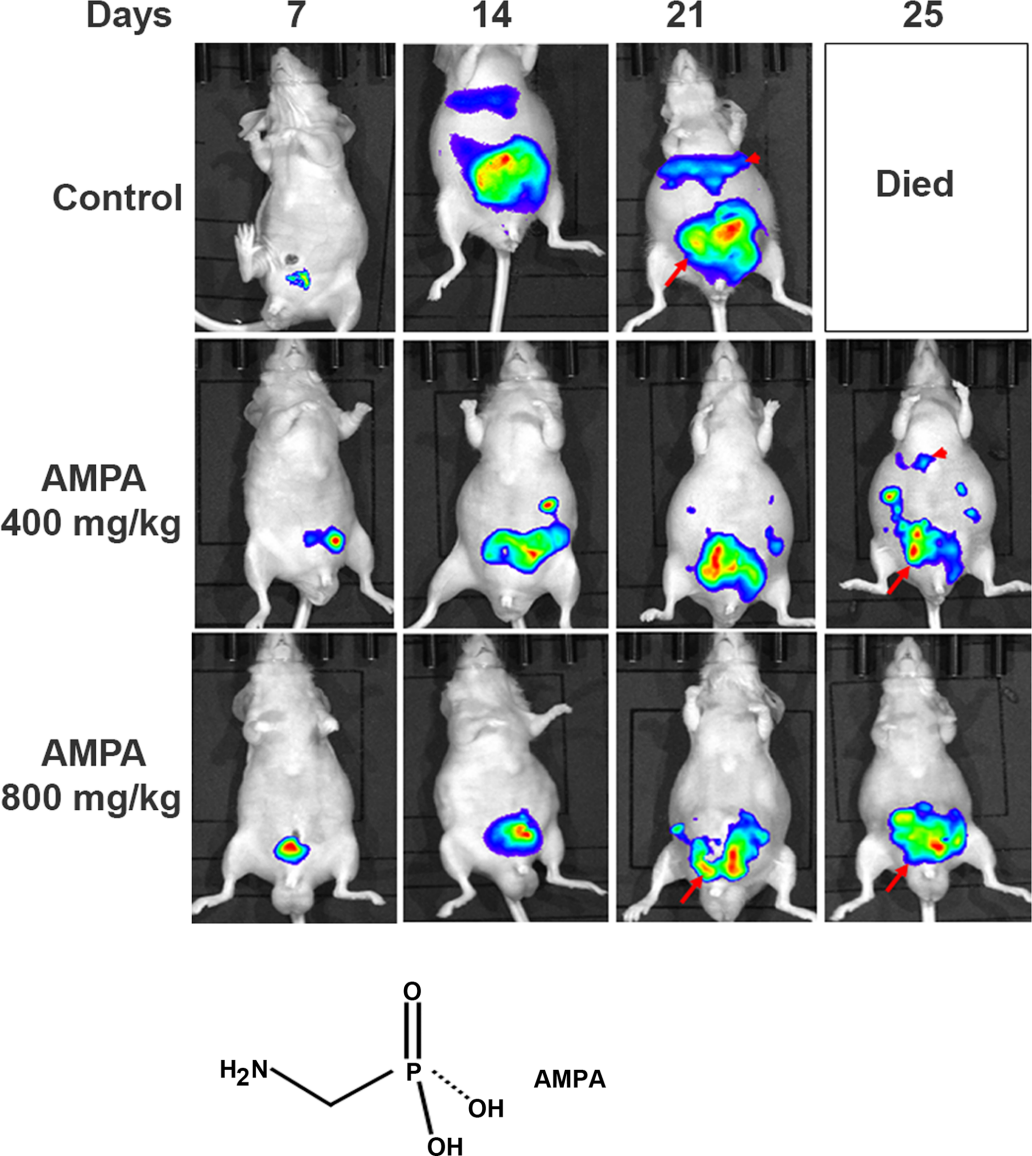
Non-invasive monitoring of orthotopic xenograft human prostate cancer in athymic nude mice Representative bioluminescence images of the anesthetized mice from the control and AMPA-treated groups are shown. The days indicate the time of bioluminescence imaging after tumor implantation and the daily treatment started at day 7. Arrows indicate the primary prostate tumors and arrowheads indicate liver and other intraabdominal metastases. The chemical structure of AMPA is present.

**Figure 2:**
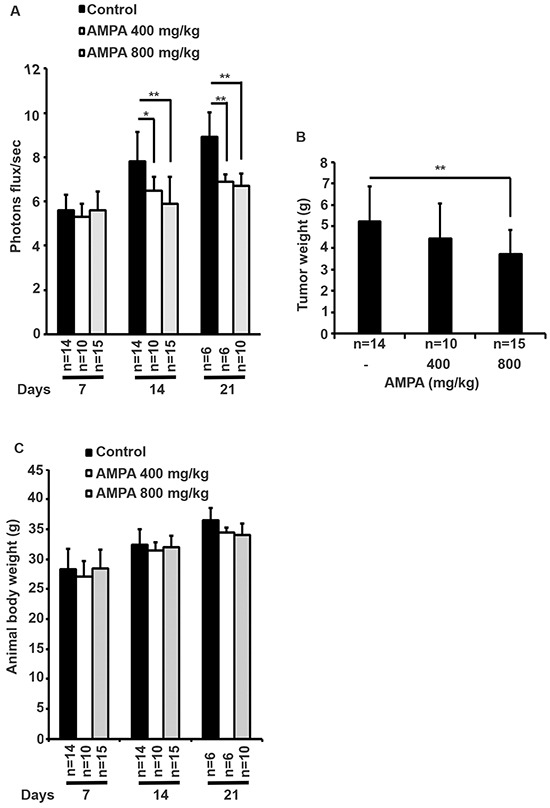
Effects of AMPA treatment on prostate tumor growth in athymic nude mice **A.** The bioluminescence values (photons flux/sec) of the prostate region were quantified for each group of mice and the mean ± SEM (error bars) are plotted over time. The number of animals in each group was shown under the X-axis, which is decreased during the time course due to animal deaths. **B.** The prostate tumor weights at necropsy. Data represent the mean ± SEM with the number of animals shown under the X-axis. *p<0.05 and **p<0.01, compared to the control group. **C.** Animals' body weight was monitored every 4-7 days. Data represent the mean ± SEM with the indicated animal number. There is not any significant difference among the control, low dose and high dose groups during the course of treatment (*p*>0.05).

We used human prostate cancer PC-3-LacZ-luciferase cells that stably expressed luciferase gene, so that the tumors could be monitored in live mice using IVIS^®^ Lumina XRMS bioluminescence imaging system (PerkinElmer, Waltham, MA, USA). PC-3-LacZ-luciferase cells were first injected subcutaneously into the flanks of athymic nude male mice. After three weeks, subcutaneous tumors were harvested and surgically implanted into the ventral lateral lobes of the prostates of 39 athymic nude male mice. Seven days later, mice were randomized into three groups: 1) control group (*n* = 14, treated intraperitoneally with 0.2 ml phosphate buffered saline); 2) low dose group (*n* = 10, treated intraperitoneally with AMPA at 400 mg/kg body weight/day); and 3) high dose group (*n* = 15, treated intraperitoneally with AMPA at 800 mg/kg body weight/day). The rationale to choose these doses was that the previous toxicity studies had shown that 400 mg/kg/day of AMPA did not cause any adverse effects and 1200 mg/kg/day decreased body weight gain in rodents, thus a maximum of 800 mg/kg/day was used as high dose. The doses are achievable in animals through intraperitoneal injection and may be achievable in humans through the same route of administration or through intravenous infusion, thus the doses are clinically relevant. Treatment was given daily up to the endpoints (animal deaths). The tumors were monitored every 4-7 days using bioluminescence imaging (Figure 1). We found that all of the mice in the control group were dead approximately three weeks after tumor implantation due to large primary tumors and metastases, while some mice in the AMPA treatment groups survived up to 35 days. The tumor sizes (represented by the peak photons flux per second) were not significantly different among the three groups at day 7 when the treatment was started. The tumor sizes of the two AMPA treatment groups were significantly smaller than that of the control group at days 14 and 21, respectively (Figure 2A). At necropsy, the average tumor weight of the high dose group was significantly less than that of the control group (Figure 2B). The average tumor weight of the low dose group was slightly less than that of the control group, but the difference was not statistically significant (Figure 2B). We monitored animals' body weight every 4-7 days and found that there was not any significant difference among the control, low dose and high dose groups during the course of treatment (Figure 2C, *p*>0.05). The histology of major organs including the brain, heart, lungs, liver, kidneys, and prostate, did not show any differences among the three groups (data not shown).

### AMPA treatment prolongs the survival time of the mice bearing human prostate tumors

**Figure 3:**
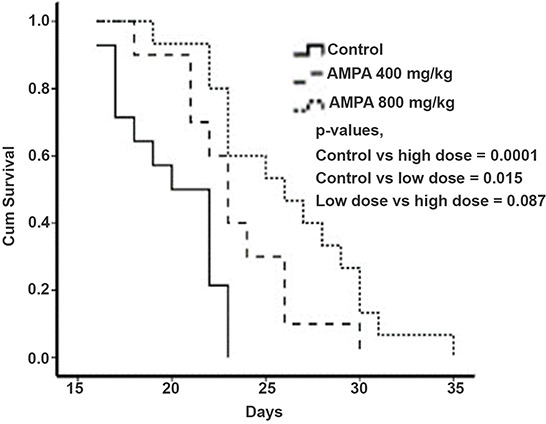
AMPA treatment prolongs the survival time of mice with prostate tumors Kaplan-Meier survival curves of mice treated without or with low and high doses of AMPA are shown. *P* values from each comparison are presented.

We found that the median survival time of the control group was approximately 20 days, whereas the median survival time of the low and high dose groups was approximately 24 and 26 days, respectively. Kaplan-Meier curves showed that the survival time of the AMPA treatment groups was significantly longer than that of the control group (Figure 3).

### AMPA treatment inhibits prostate tumor metastasis

**Figure 4:**
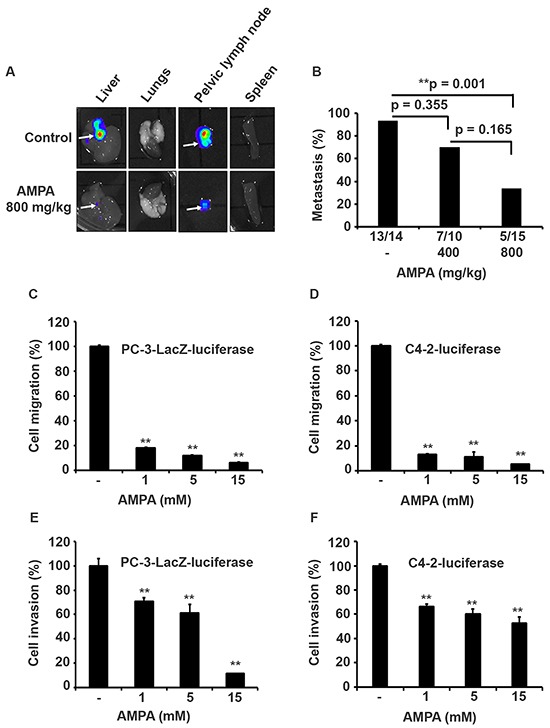
AMPA treatment inhibits prostate cancer metastasis *in vivo* and cell migration/invasion *in vitro* **A.** Representative bioluminescence images of the excised liver, lungs, pelvic lymph node, and spleen from the control and high dose groups. Arrows indicate the organs with metastasis as identified by bioluminescence imaging. **B.** Percentage of metastasis from each group is shown on the Y-axis and the actual number of animals with metastases is shown under the X-axis. **C–F.** Effects of AMPA on cell migration and invasion. PC-3-LacZ-luciferase and C4-2-luciferase cells were plated in Transwell^®^ plates and treated with AMPA at concentrations of 1, 5, and 15 mM for 24 h in migration assays and 48 h for invasion assays as described in Materials and Methods. The quantification of cell migration and invasion was normalized to the control group (without AMPA treatment); **p<0.01, compared to the control group.

Bioluminescence imaging detected bioluminescent signals out of the area of mouse prostates, indicating tumor metastases (Figure 1). To confirm which organs had metastatic tumors, we excised the liver, lungs, pelvic lymph nodes, and spleen, and did ex vivo bioluminescence imaging. We found that the control group had high incidence of metastasis into the liver and pelvic lymph nodes (Figure 4A). The incidence of metastasis was decreased in the AMPA-treated mice, particularly in the high dose group (Figure 4B). To determine whether AMPA could affect cell migration and invasion, we performed cell migration assays using Transwell^®^ plates and cell invasion assays using basement membrane-coated Transwell^®^ plates. The cancer cells were treated without or with 1, 5, and 15 mM AMPA for 24 h in migration assays and 48 h in invasion assays. As shown in Figure 4C-4D, AMPA at doses of 1, 5, and 15 mM effectively decreased migration of both PC-3-LacZ-luciferase and C4-2-luciferase cells. The migrated PC-3-LacZ-luciferase cell number was 18.4% (with 1 mM AMPA), 12.2% (with 5 mM AMPA), and 6.2% (with 15 mM AMPA) of the untreated control group. The migrated C4-2-luciferase cell number was 12.9% (with 1 mM AMPA), 10.94% (with 5 mM AMPA), and 5.2% (with 15 mM AMPA) of the untreated control group (Figure 4C-4D, *p*<0.01 compared to the control group). Similarly, AMPA at doses of 1, 5, and 15 mM effectively decreased invasion of basement membrane in both PC-3-LacZ-luciferase and C4-2-luciferase cells. The invaded-through PC-3-LacZ-luciferase cell number was 71% (with 1 mM AMPA), 61.2% (with 5 mM AMPA), and 11.4% (with 15 mM AMPA) of the untreated control group. The invaded-through C4-2-luciferase cell number was 66.3% (with 1 mM AMPA), 60.1% (with 5 mM AMPA), and 52.4% (with 15 mM AMPA) of the untreated control (Figure 4E-4F, *p*<0.01 compared to the control group). Of note, AMPA inhibition of *in vivo* metastasis appears to be dose-dependent (Figure 4B), whereas the *in vitro* cell migration and invasion assays (Figure 4C-4F) appear dose-independent except PC-3-LacZ-luciferase cell invasion (Figure 4E) that is dose-dependent. This reflects the differences between *in vivo* and *in vitro* conditions. At least, the dose-dependent inhibition of PC-3-LacZ-luciferase cell invasion is consistent with the dose-dependent inhibition of metastasis.

### AMPA treatment alters expression of apoptotic and cell cycle regulatory genes

**Figure 5:**
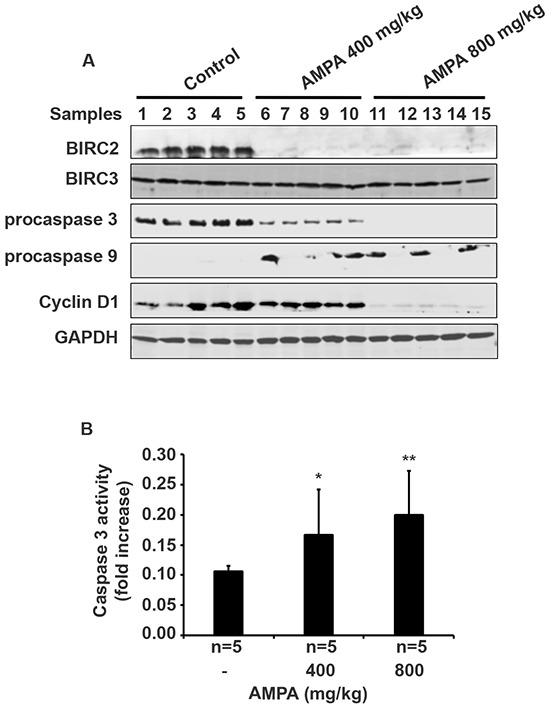
AMPA treatment changes gene expression in the prostate tumors **A.** Five prostate tumors from each group were randomly selected for Western blot analysis of protein expression. **B.** Caspase 3 activity was assessed and data represented the mean ± SEM (*n* = 5). *p<0.05 and **p<0.01, compared to the control group (without AMPA treatment).

To study the genes involved in apoptosis, we did Western blot analysis of the protein expression in the tumors treated without or with AMPA at low and high doses. As shown in Figure 5A, AMPA at low and high doses decreased the protein expression of Baculoviral IAP Repeat Containing 2 (BIRC2, also called cIAP1), but did not change the expression of BIRC3 (also called cIAP2) proteins. AMPA treatment elicited a dramatic decrease of procaspase 3 protein at the low dose and essentially abolished procaspase 3 protein at the high dose, compared to the untreated control tumors (Figure 5A). Decrease of procaspase 3 indicates cleavage of procaspase 3 into active caspase 3, a key executioner caspase in apoptosis [11]. To confirm if caspase 3 was activated in the tumors, we performed caspase 3 activity assays. We found that caspase 3 activity was significantly increased by 1.57- and 1.89-fold in the low and high dose groups, respectively, compared to the untreated control group (Figure 5B, *p*<0.05 or 0.01). Further, procaspase 9 protein levels were increased in the AMPA-treated tumors, compared to the untreated tumors (Figure 5A). In addition, we also observed a dramatic decrease of cyclin D1 protein levels in the tumors treated with high dose of AMPA (Figure 5A). This change was specific for cyclin D1 as cyclin D3 levels were almost undetectable in the tumors (data not shown).

### AMPA treatment decreases proliferation, increases apoptosis, and reduces angiogenesis

**Figure 6:**
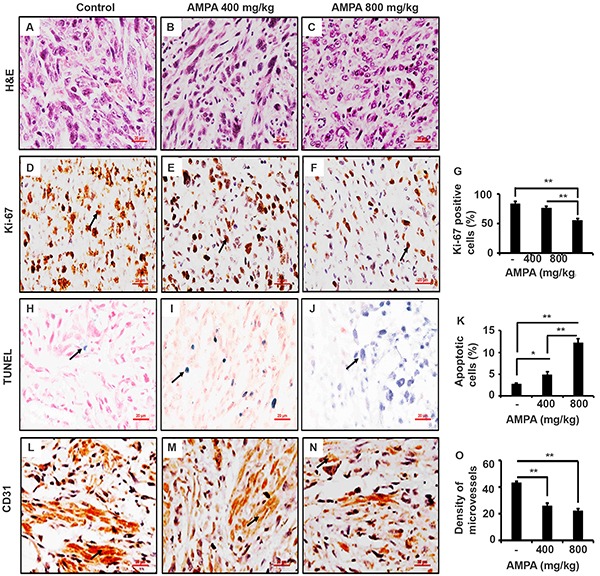
AMPA treatment inhibits tumor cell proliferation, increase tumor cell apoptosis, and reduces density of microvessels in the prostate tumors **A-C.** Representatives of H&E stained prostate tumor sections from each group. **D-F.** Ki-67 staining; arrows indicate the positive cells. **G.** Percentage of Ki-67 positive cells counted in five high-power fields. **H-J.** TUNEL staining; arrows indicate the positive cells. **K.** Percentage of apoptotic cells was counted in five high-power fields. **L-N.** Representatives of CD31 staining; arrows indicate microvessels. **O.** The number of microvessels was counted in five high-power fields. Original magnification, x400; scale bars, 20 μm. *p<0.05 and **p<0.01, compared to the control group.

We performed histopathological examination of the primary tumors derived from PC-3-LacZ-luciferase cells and confirmed that the tumors contained cells with typical malignant features (Figure 6A-6C). Immunohistochemical (IHC) staining showed that the Ki-67-positive cell number was slightly decreased in the tumors treated with low dose AMPA, however, the Ki-67-positive cell number was significantly decreased in the tumors treated with high dose AMPA, compared to the untreated tumors (Figure 6D-6G, *p*<0.01). On the other hand, the number of apoptotic cells as detected by terminal deoxynucleotidyl transferase dUTP nick end labeling (TUNEL) staining was significantly increased in the tumors treated with both low and high doses of AMPA, compared to the untreated tumors (Figure 6H-6K, *p*<0.05 or 0.01). Further, we evaluated the density of microvessels in the tumors by IHC staining of CD31. We found that the density of microvessels was significantly decreased in the tumors treated with both low and high doses of AMPA, compared to the untreated tumors (Figure 6L-6O, *p*<0.01).

## DISCUSSION

In the present study, we demonstrated that AMPA inhibited tumor growth and metastasis in an orthotopic xenograft mouse model of prostate cancer [12]. We showed that the tumor size was smaller in the AMPA-treated mice compared to the untreated control mice. The AMPA-treated mice survived longer than the untreated control mice. AMPA treatment decreased the number of metastatic tumors in the liver and pelvic lymph nodes. The anti-metastatic property of AMPA may be due to AMPA's function in inhibiting cancer cell migration and invasion. These findings clearly demonstrate that AMPA has anti-cancer function. Of note, the high-dose group had smaller primary tumor and a lower rate of metastases at the endpoint compared to the control group, which raised a question whether the tumor burden was the key reason that caused death in the high-dose group. Our answer to this question is that the tumor burden is still the key reason for animal death, because the reduced tumor burden in the high-dose group is relative to the control group at the time point for comparison. As the high-dose animals survived longer time, the tumors continued to grow in size and in number of metastases, which eventually led to animal death.

How AMPA exerts its function has been shown in our previous *in-vitro* studies [9, 10]. We demonstrated previously that AMPA up-regulated p53 and p21 as well as procaspase 9, activated caspase 3, and down-regulated cyclin D3 in C4-2B cells [9]. AMPA and particularly AMPA in combination with methoxyacetic acid down-regulated BIRC2 expression in PC-3 cells [10]. Therefore, we assessed the expression levels of the aforementioned genes in the tumors. Consistently, we found that BIRC2 protein expression was abolished in the tumors treated with low or high dose of AMPA. It appears that AMPA specifically decreased BIRC2 expression, as BIRC3 levels were not changed by AMPA treatment. The exact molecular mechanisms of how AMPA decreases expression of BIRC2 and cyclin D are not known. We speculate that AMPA may cause stress responses in the cancer cells due to interruption of peptide synthesis (because AMPA is a glycine analog) and/or nucleic acid synthesis (because glycine provides the central C_2_N subunit of all purines). Overexpression of BIRC2 has been shown to suppress apoptosis induced by a variety of stimuli [13–16]. BIRC2 has been shown to bind and potently inhibit caspase 3 [17, 18]. Here we found that AMPA treatment led to cleavage of procaspase 3 and increase of caspase 3 activities. Since caspase 3 is a key executioner caspase for apoptosis [11], it is not surprising that we found an increase in apoptotic cells in the tumors treated with AMPA. Thus, our findings suggest that AMPA decreases BIRC2 expression to activate caspase 3 and then induce apoptosis in the cancer cells. We also observed an increase of procaspase 9 levels by AMPA treatment, which is consistent to our findings in *in-vitro* studies [9, 10]. Increased procaspase 9 level may increase formation of Apaf-1/procaspase 9 complex to initiate apoptosis as shown by Sakai et al [19].

We found that cyclin D1 levels were dramatically decreased in the tumors treated with high dose of AMPA, but not much in the tumors treated with low dose of AMPA. This difference at molecular levels is consistent with the difference in tumor cell proliferation, that is, only high dose AMPA significantly decreased cell proliferation as shown by Ki-67 staining. Cyclin D1 is a key regulator of G1-to-S phase transition of the cells. Overexpression of cyclin D1 is associated with an oncogenic event in androgen-independent metastatic prostate cancer, suggesting a link of cyclin D1 expression to metastasis [20]. Comstock et al. [21] reported that nuclear localization of cyclin D1 is associated with lymph node metastasis of prostate cancer. Cyclin D1 has been shown to promote cellular motility through inhibiting Rho-activated kinase signaling and repressing the metastasis suppressor thrombospondin 1 [22]. Thus, AMPA inhibits prostate cancer metastasis through suppressing cyclin D1 expression, implicating that AMPA may be used for prevention of prostate cancer metastasis. Deprivation of extracellular glycine slowed down fast proliferating cancer cells by prolonging the G1 phase of the cell cycle [7]. Our findings suggest that AMPA inhibits cell proliferation through decreasing cyclin D1 expression.

Angiogenesis is a physiologic process involving the growth of new blood vessels from preexisting vessels and is required for tumor growth and metastasis [23]. The growth of solid tumors depends on angiogenesis and suppression of angiogenesis offers an option for the treatment of cancer [24]. We found that AMAP reduced the density of microvessels in the tumors, which indicates that AMPA may also inhibit angiogenesis.

In summary, we have demonstrated that AMPA inhibits growth and metastasis of orthotopically xenografted human prostate cancer and prolongs the survival of animal hosts. AMPA specifically decreases BIRC2 expression to activate caspase 3, leading to apoptosis of the tumor cells. AMPA decreases cyclin D1 to inhibit tumor cell proliferation and metastasis. AMPA also inhibits angiogenesis. Our findings suggest that AMPA may be a potential anticancer agent in the treatment of human prostate cancer.

## MATERIALS AND METHODS

### Mice

Animal protocol was approved by the Animal Care and Use Committee of Tulane University in accordance to the National Institutes of Health Guidelines for the Care and Use of Laboratory Mice. Six to eight-weeks-old Ncr-nu/nu male mice were purchased from the National Cancer Institute Animal Production Program and were used for subcutaneous and surgical orthotopic transplantation of PC-3-LacZ-luciferase cells or fresh xenograft tumor tissues. All of the mice were maintained in a specific pathogen-free environment. All of the mice were maintained in a daily cycle of 12 hour (h) light and 12 h darkness. PC-3-LacZ-luciferase cells were injected subcutaneously in both flanks of five mice. Three weeks later, fresh tumor tissues were harvested from these mice. The tumor tissues from the periphery of the subcutaneous tumors were cut into small cubes of approximately 1 mm^3^ in phosphate-buffered saline (PBS). The tumor pieces were randomly mixed and an equal amount of 5 pieces were implanted in each mouse prostate as described [25]. A total of 39 mice were anesthetized with 2-4% isoflurane inhalation. The skin over the lower abdomen was gently cleaned prior to disinfection by 70% ethanol and then followed by Betadine solution. A 0.5 cm incision was made right above symphysis pubis to expose the prostate gland. The fascia surrounding the ventral portion of the prostate was carefully isolated and the two ventral lateral lobes of the gland were separated by a small incision using a pair of fine surgical scissors. Five tumor pieces were sutured into the incision using an 8-0 nylon suture. The two parts of the separated lobes of the prostate gland were then sutured together with the five tumor pieces wrapped within. The surrounding fascia was then used to wrap this portion of the gland. The testes were exposed by pulling the epididymal adipose tissue. A hemostat was applied to curtail blood flow followed by ligation with 6-0 sutures and removal of the testes. Buprenex (0.1 mg/kg) was given subcutaneously as analgesics at the end of surgical procedure and then every 12 h up 48 h.

### Treatment and bioluminescence imaging

One week after orthotopic tumor implantation, the tumor size was monitored by non-invasive bioluminescent imaging, using IVIS^®^ Lumina XRMS Series III (PerkinElmer, Waltham, MA, USA). Then, the mice were stratified according to the tumor size and randomized into three groups bearing tumors with equal average sizes (i.e., there was no statistically significant difference). The three groups were: 1) control group (*n* = 14, treated intraperitoneally with 0.2 ml PBS); 2) low dose group (*n* = 10, treated intraperitoneally with AMPA at 400 mg/kg body weight/day); and 3) high dose group (*n* = 15, treated intraperitoneally with AMPA at 800 mg/kg body weight/day). Treatment was given daily up to the endpoints (animal deaths). The tumors were monitored every 4-7 days using bioluminescence imaging. Briefly, 200 μl PBS containing 3 mg substrate D-luciferin sodium salt (Gold Biotechnology, St Louis, MO, USA) was injected intraperitoneally in each mouse prior to imaging. Image acquisition started immediately with a series of images taken once every two minutes up to 30 minutes, thus determining the peak light emission. One second (sec) exposure was acquired (Emission filter: open f/stop: 1, binning: medium). Region of interest (ROI) of the same size and shape was used for all mice throughout the study. The bioluminescence images were quantified by measuring the total photons over the prostate region and the average photon flux within the ROI was calculated as photon flux/sec, using the vendor's software (Living Image^®^ 4.0, PerkinElmer, Waltham, MA, USA). Bioluminescence imaging was also performed in the excised liver, lungs, pelvic lymph nodes, and spleen at necropsy. The animals were observed to their natural death or were euthanized when the veterinarian determined that they were terminally sick with no more than 12 hours of survival time.

### Western blot analysis

A portion of the excised prostate tumor tissues from 5 randomly selected mice per group was used to prepare whole tissue lysates. Briefly, prostate tissues were sonicated in radioimmunoprecipitation assay (RIPA) lysis buffer (50 mM sodium fluoride, 0.5% Igepal^®^ CA-630 [NP-40], 10 mM sodium phosphate, 150 mM sodium chloride, 25 mM Tris pH 8.0, 1 mM phenylmethylsulfonyl fluoride, 2 mM ethylenediaminetetraacetic acid [EDTA], 1.2 mM sodium vanadate) supplemented with protease inhibitor cocktail (Sigma-Aldrich Corp, St Louis, MO, USA). The lysates were centrifuged at 14,000 rpm for 15 min at 4°C. Supernatants (proteins) were collected and stored at −80°C until further use. Fifty μg of proteins were subjected to 10% sodium dodecyl sulfate-polyacrylamide gel electrophoresis and transferred to polyvinylidene difluoride membranes (Bio-Rad Laboratories, Hercules, CA, USA). The membranes were blocked with 5% nonfat dry milk in TBST buffer (25 mM Tris-HCl, 125 mM NaCl, 0.1% Tween 20) for 1 hour and probed with the indicated primary antibodies overnight and then IRDye^®^800CW- or IRDye®680-conjugated secondary antibodies (LI-COR Biosciences Inc, Lincoln, NE, USA) for 1 hour. The results were visualized using an Odyssey^®^ Infrared Imager (LI-COR Biosciences Inc). For loading control, the membranes were stripped and probed for glyceraldehyde-3-phosphate dehydrogenase (GAPDH). The antibodies used were: rabbit anti-procaspase 9 and mouse anti-cyclin D3 antibodies were purchased from Cell Signaling Technology (Danvers, MA, USA). Rabbit anti-BIRC2 and rabbit anti-BIRC3 antibodies were obtained from Santa Cruz Biotechnology (Dallas, TX, USA). Mouse anti-GAPDH and mouse anti-procaspase 3 antibodies were purchased from EMD Millipore Corp (Billerica, MA, USA). Rabbit anti-cyclin D1 antibodies were purchased from Abcam (Cambridge, MA, USA).

### Caspase 3 activity assay

Caspase 3 activity assay was measured with ApoAlert^®^ Caspase 3 Colorimetric Assay Kit (Clontech Laboratories, Inc., Mountain View, CA, USA) according to the manufacturer's instructions. Briefly, the prostate tumor tissues were lysed and centrifuged. The supernatant was analyzed for protein concentration and 200 μg of protein in a 50 μl volume was mixed with 50 μl of 2x reaction buffer and 5 μl of 4 mM DEVD-pNA substrate was added to produce a final substrate concentration of 200 μM. After 90-minutes incubation at 37°C, absorbance was measured at 405 nm using a plate reader (Bio-Tek U.S., Winooski, VT, USA). Fold increase in caspase 3 activity was determined by comparing the results of treated samples with the level of the untreated control.

### Migration and invasion assay

PC-3-LacZ-luciferase and C4-2-luciferase cells (stably expressing luciferase gene transfected into human prostate cancer cell lines) were trypsinized and resuspended in serum free medium. In migration assays, the Transwell^®^ plates (Corning Life Sciences, Corning, NY, USA) used were the product #3422 with 8.0-μm pore polycarbonate membranes as inserts, and in invasion assays, the Transwell^®^ inserts were coated with Culture^®^ basement membrane (product #3458). Approximately 2×10^5^cells/well were placed in the upper chamber of Transwell^®^ inserts and treated with AMPA at concentration of 1, 5, and 15 mM. The control group was treated with PBS. The lower chamber was filled with medium containing 10% fetal bovine serum as chemoattractant. Each group had triplicate wells. After 24 (in migration assays) and 48 h (in invasion assays), the cells remained (not migrated or invaded) in the upper chamber were removed with cotton-tipped applicators. The cells that migrated or invaded to the lower chamber were lysed with 300 μl cell lysis buffer. Luciferase activity of 100 μl of the cell lysates was determined using Bright-Glo™ Luciferase Assay System (Promega, Madison, WI, USA) in an Optocomp II luminometer (MGM Instruments, Inc., Hamden, CT, USA). Because the cells stably expressed luciferase genes, the luciferase signals were proportional to the number of the cells migrated or invaded through the inserts. The AMPA-treated groups were normalized to the control group (without AMPA treatment).

### Immunohistochemical and terminal deoxynucleotidyl transferase-mediated dUTP nick end labeling (TUNEL) staining

At necropsy, the prostate tumors were dissected out and weighed. Tumor tissues and major organs were fixed in 4% paraformaldehyde, embedded in paraffin, and cut into 4-μm-thick sections. Hematoxylin & eosin (H&E) staining was performed to evaluate tissue morphology. Ki-67 and CD31 immunohistochemical staining was performed as described previously [26, 27]. The antibodies and reagents used were: rabbit anti-Ki-67 (1:200 dilution, EMD Millipore, Billerica, MA, USA); rabbit anti-CD31 (Ab28364, 1:50 dilution, Abcam, Cambridge, MA, USA); and Vectastain^®^ ABC kit (Vector Laboratories, Burlingame, CA, USA). Terminal deoxynucleotidyl transferase-mediated dUTP nick end labeling (TUNEL) staining was performed using TACS. XL^®^ Blue Label *In Situ* Apoptosis Detection Kits (Trevigen, Inc., Gaithersburg, MD, USA) according to the manufacturer's instructions [26]. To quantify Ki-67-positive and TUNEL-positive cells, three prostate tumors from each group were randomly selected and stained. Approximately 200 cells per field of five high-power fields (x400 magnification) of each prostate tumor were counted and the percentages of positive cells were calculated as the number of positive cells divided by the total number of cells. The density of micro-vessels was evaluated by counting the CD31-positive microvessels in five high-power fields per tumor and the average number of CD31-positive microvessels per high-power field in three random mouse prostate tumors per group was compared.

### Statistical analysis

The normality of the bioluminescence imaging data was assessed and the log-transformed values were found to conform to normality assumptions better than raw values. Thus, subsequent analysis used the log-transformed values. The quantitative data were presented as the mean ± standard error of the mean (SEM) and analyzed with the Student's *t* test (two-tailed). Animal survival time was determined utilizing Kaplan-Meier survival analysis and the log-rank test. Differences in metastases were analyzed using Chai-square test. A *p*-value < 0.05 was considered statistically significant.
